# Roles of Interleukin-1 Receptor Antagonist in Prostate Cancer Progression

**DOI:** 10.3390/biomedicines8120602

**Published:** 2020-12-13

**Authors:** Yu-Ching Fan, Kuan-Der Lee, Yuan-Chin Tsai

**Affiliations:** 1 PhD Program for Cancer Molecular Biology and Drug Discovery, College of Medical Science and Technology, Taipei Medical University and Academia Sinica, Taipei 110301, Taiwan; d621109001@tmu.edu.tw; 2 Department of Hematology and Oncology, Taipei Medical University Hospital and Department of Medicine, Taipei Medical University, Taipei 110301, Taiwan; kdlee@h.tmu.edu.tw; 3 PhD Program for Cancer Molecular Biology and Drug Discovery, College of Medical Science and Technology, Taipei Medical University, Taipei 110301, Taiwan; 4 Graduate Institute of Cancer Biology and Drug Discovery, College of Medical Science and Technology, Taipei Medical University, Taipei 110301, Taiwan

**Keywords:** interleukin-1 receptor antagonist, tumor microenvironment, tumor-infiltrating leukocytes, castration-resistant prostate cancer

## Abstract

Background: Inflammation is known to promote tumor formation and progression; however, we found a natural anti-inflammatory factor, interleukin (IL)-1 receptor antagonist (IL1RN), in a mouse transgenic adenocarcinoma of the mouse prostate (TRAMP)-C1-derived tumor microenvironment (TME). We sought to characterize the functions of the IL1RN-secreting cells in the TME. Methods: We compared tumors collected from two syngeneic mouse models and isolated tumor-infiltrating leukocytes (TILs) with different cluster of differentiation 11b (CD11b) statuses. We examined the proliferation functions of the TILs and the IL1RN using several approaches, including a colony-formation assay and DNA synthesis levels. Results: We demonstrated that CD11b-deficient TILs (TILs/CD11b^−^) secreted the IL1RN and promoted proliferation by analyzing conditioned media. In addition to mouse TRAMP-C1, proliferation functions of the IL1RN were confirmed in several human castration-resistant prostate cancer (CRPC) cell lines and one normal epithelial cell line. The androgen-sensitive lymph node carcinoma of the prostate (LNCaP) cell line showed cytotoxic responses to IL1β treatment and androgen-dependent regulation of IL-1 receptor type 1 (IL1R1), while the C4-2 CRPC cell line did not. IL1RN rescued LNCaP cells from the cytotoxic effects of IL1β/IL1R1 signaling. Conclusions: Our results support TILs/CD11b^−^ cells being able to protect androgen-dependent cells from inflammatory damage and promote the malignant progression of prostate cancers partly through the IL1RN in the TME.

## 1. Introduction

Targeting activities of the androgen receptor (AR) by depriving it of androgen is a key therapeutic approach for prostate cancer; however, resistant tumors eventually occur and can progress to metastatic castration-resistant prostate cancer (CRPC) [1]. Multiple types of mutations are selected for in CRPC to overcome the inhibitory effects of androgen-deprivation therapy (ADT) [2]; however, a key driver of the malignant progression is the inflammatory environment [3,4,5]. Inflammation is known to promote tumor formation and progression. Mice with a deficiency of either tumor necrosis factor (TNF)-α, a master proinflammatory cytokine, or C-C motif chemokine ligand 2 (CCL2), a proinflammatory chemokine, were refractory to the development of malignant tumors in the classical two-stage skin carcinogenesis model [6]. Several key cytokines, such as TNF-α and interleukin-1β (IL1β), stimulate inflammatory programs that coordinate functions of tumor-infiltrating leukocytes (TILs) and their presence in the tumor milieu via a plethora of proinflammatory chemokines [7]. Thus, dynamic interactions between tumor cells and TILs in the inflammatory tumor microenvironment (TME) facilitate development of CRPC [4,5].

The IL-1 receptor antagonist (IL1RN) belongs to the IL-1 family and competes with IL-1 ligands (e.g., IL1β) for their receptor, IL-1 receptor type 1 (IL1R1); however, due to a lack of interaction with the co-receptor, IL-1 receptor accessory protein (IL1RAP), the IL1RN does not lead to stimulation of inflammatory signaling [8]. Therefore, the IL1RN serves as a natural anti-inflammatory cytokine, and was shown to have therapeutic benefits in many types of inflammatory disease models [9]. Consistent with the roles of inflammation in tumor promotion, the IL1RN was reported to be an antitumor agent partly through inhibiting IL1R1 signaling [10,11]. However, emerging evidence also supports a tumor-promoting role of the IL1RN. It was reported that the IL1RN increased the proliferation of prostate epithelial cells [12]; also, the IL1RN was shown to antagonize oncogene-induced senescence in a *Pten*-null prostate cancer model [13] and was involved in the growth of pancreatic intraepithelial neoplasias in a *Kras*-G12D-mutant pancreatic cancer model [14]. These seemingly contrasting results may reflect the complicated nature of inflammation in the TME. It is also possible that different experimental settings (systemic administration vs. genetic models) affect the physiological statuses and, consequently, outcomes in the TME. Thus, the role of the IL1RN in tumor biology remains unclear.

In this study, we have analyzed the TME in two syngeneic cancer models (Lewis lung carcinoma, LLC1, vs. the transgenic adenocarcinoma of the mouse prostate (TRAMP)-C1 cell line) [15,16]. Our results show that TILs with a cluster of differentiation 11b (CD11b)-deficient population (TILs/CD11b^−^) contribute to an anti-inflammatory environment partly through secretion of the IL1RN. The IL1RN can promote cell proliferation and prevent IL1β-induced cell death, which may facilitate development of CRPC.

## 2. Experimental Section

### 2.1. Cell Lines and Analyses of the TME

The mouse cell lines (tumorigenic TRAMP-C1 and Lewis lung carcinoma, LLC1) and human prostate cancer cell lines (C4-2, DU145, PC3, LNCaP, and RWPE-1) were purchased from the American Type Culture Collection (ATCC; Manassas, VA, USA). All cells were maintained in culture medium suggested by the ATCC. For dihydrotestosterone (DHT) treatment, cells were starved for 24 h in a 10% charcoal-stripped serum (CSS)-containing RPMI 1640 medium (Invitrogen, Carlsbad, CA, USA) followed by incubation with 10 nM DHT (Sigma-Aldrich, St. Louis, MO, USA) for 2 days. In experiments with an AR inhibitor (MDV3100 (MDV)), cells were incubated with 10 µM MDV3100 (Selleckchem, Houston, TX, USA) for 24 h in 10% serum-containing an RPMI 1640 medium.

To analyze gene expressions in the TME, the collected tumors derived from the TRAMP-C1 and LLC1 cell lines in syngeneic C57BL/6 mice were dissected and immediately frozen in liquid nitrogen. Then, samples were processed for a reverse-transcription (RT) quantitative polymerase chain reaction (qPCR). To analyze the cytokine/chemokine profiles in the TME, collected tumors were incubated with collagenase type IV (10 ng/mL, Gibco, Grand Island, NY, USA) and DNase type IV (200 U/mL, Sigma-Aldrich), and then filtered through a nylon mesh with a 100-μm pore size (Falcon Cell Strainers, Thermo Fisher Scientific, Waltham, MA, USA) to obtain single-cell suspensions. Red blood cells were removed by ACK lysis buffer (Thermo Fisher Scientific). Cells were seeded in culture dishes, and conditioned media of most leukocytes and tumor cells were collected for cytokine/chemokine analyses using an antibody array (Proteome Profiler™ Array, R&D System, Minneapolis, MN, USA).

To prepare the CD11b-positive (CD11b^+^) and CD11b-negative (CD11b^−^) leukocytes, single-cell suspensions from either splenocytes or tumors were first processed using discontinuous density gradient Ficoll-Paque Plus density gradient media (GE Healthcare Life Sciences, Uppsala, Sweden) to derive the normal leukocytes or TILs. In brief, a single-cell suspension was re-suspended in phosphate-buffered saline (PBS) and carefully layered onto a Ficoll solution. Following centrifugation (at 400× *g* for 30 min), normal leukocytes or TILs in the intersection were collected. Cells with different CD11b statuses were separated using an EasySep™ mouse CD11b-positive selection kit II (STEMCELL Technologies, Vancouver, BC, Canada).

### 2.2. RT-qPCR

Total RNA was isolated from different types of samples followed by TRIzol (Invitrogen) and the RNA isolation system (Qiagen, Venlo, The Netherlands). For complimentary (c)DNA preparation, 1 µg of total RNA was used for RT (Invitrogen). The amplification step was performed and measured with a SYBR Green PCR master mix (Applied Biosystems, Beverly, MA, USA). For all primer pairs, the thermocycler was run for an initial 95 °C for 10 min, followed by 40 cycles of 95 °C for 15 s and 60 °C for 1 min. All reactions were normalized to mouse *Gapdh* and analyzed in triplicate. All primers used are listed in Table S1.

### 2.3. Syngeneic Prostate Cancer Model

Animal experiments were performed in accordance with a protocol approved by the Taipei Medical University Animal Care and Use Committee (no.: LAC-2017-0274, Taipei, Taiwan). For the tumor growth analysis, 10^6^ TRAMP-C1 or 10^5^ LLC-1 cells were subcutaneously (s.c.) injected into 8-week-old male C57BL/6 mice (NLAC, Taipei, Taiwan). Mice treated with saline served as the reference control. Tumor sizes were quantified according to the formula: length (mm) × width (mm)^2^/2, and mice were sacrificed to collect tumors when the tumors had reached a size of ca. 900 mm^3^.

### 2.4. Colony-Formation Assay

For the regular two-dimensional (2D) culture, TRAMP-C1 cells (500 cells/well) were seeded into 12-well plates. Cells were incubated with either conditioned media (splenocytes and TILs) or selected cytokines/chemokines. After incubation for 10 days, colonies were photographed and analyzed manually or by the *ColonyArea* plug-in [17] with Image J software. A published procedure was followed for the three-dimensional (3D) culture [18]; in brief, TRAMP-C1 cells were seeded in Matrigel-coated (Corning, NY, USA) wells. The detailed procedure is summarized in Figure S1.

### 2.5. DNA Synthesis Assay

DNA was synthesized using a BrdU Cell Proliferation Assay Kit (BioVision, San Francisco, CA, USA), according to the manufacturer’s instructions. TRAMP-C1 cells were seeded into 96-well plates at 1000 cells/well and treated with the mouse IL1RN recombinant protein for 48 h. Bromodeoxyuridine (BrdU) was then added and incubated at 37 °C for 6 h before fixation. BrdU incorporated into the proliferating cells was detected with an enzyme-linked immunosorbent assay (ELISA).

### 2.6. Proliferation Assay

Cells (TRAMP-C1, LNCaP, C4-2, RWPE-1, DU145, PC3) were seeded in 96-well plates for 24 h followed by treatment with mouse or human IL1RN recombinant protein (ProSpec, Rehovot, Israel) for 3 days. Co-treatment of human IL1RN and IL1β recombinant proteins (R&D systems, Minneapolis, MN, USA) was performed similarly. A CCK-8 solution (Sigma-Aldrich) was applied to each well and incubated for 1 h. The measurement was done by reading the absorbance at 450 nm on an Epoch Microplate Spectrophotometer (BioTek Instruments, Winooski, VT, USA).

### 2.7. Western Blot Analysis

Cell lysates were processed with 6× Laemmli sample buffer followed by electrophoresis. Samples were transferred to polyvinylidene difluoride membranes and blocked with 5% milk in TBST (Sigma). Western blotting was performed using several antibodies (anti-CD11b antibody, ca. no.: A1581, ABclonal, Woburn, MA, USA; anti-IL1R1 antibody, cat. no.: PA5-47937, Invitrogen; anti-GAPDH antibody, cat. no.: GTX100118, GeneTex, Irvine, CA, USA) at 4 °C overnight. Membranes were washed with TBST twice and incubated with a horseradish peroxidase (HRP)-antibody at room temperature for 1 h. After incubation with an HRP substrate (WesternBright ECL HRP Substrate, Advansta, San Jose, CA, USA), images were taken with an Amersham™ Imager 600 (GE Healthcare, Chicago, IL, USA).

### 2.8. Statistical Analysis

All in vitro data are presented as the mean ± standard deviation (SD). Statistical calculations were performed with GraphPad Prism (GraphPad Software, CA, USA) analytical tools. *p* values of < 0.05 were considered statistically significant.

### 2.9. Abbreviations

All abbreviations used in the article are listed in Table S2 in alphabetical order.

## 3. Results

### 3.1. Confirmation of the IL1RN as a Tumor-Type-Specific Anti-Inflammatory Cytokine in the TRAMP-C1-Derived TME

**Figure 1:**
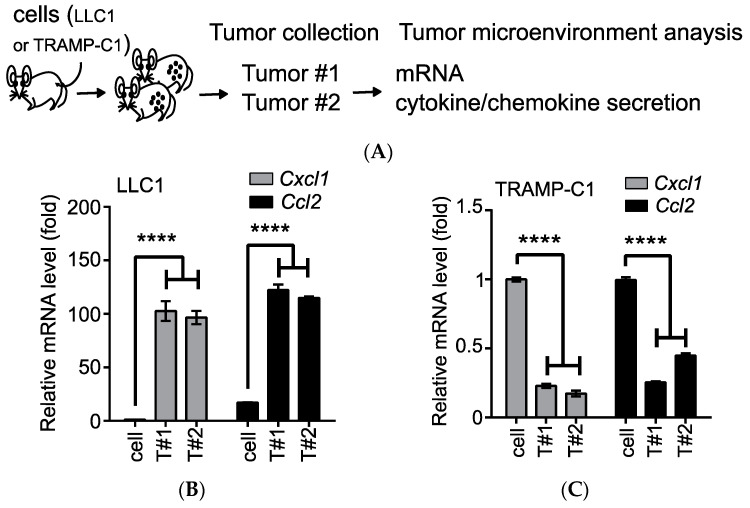

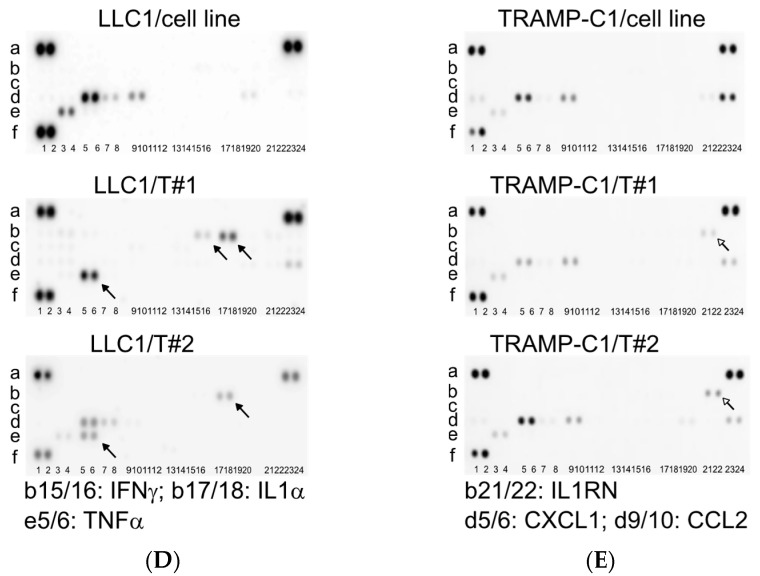
Transgenic adenocarcinoma of the mouse prostate (TRAMP)-C1-derived tumors show tumor-type-specific anti-inflammatory environments. (**A**) The outline of analyzing the tumor microenvironment. Following a subcutaneous injection, tumor tissues (Tumor#1 (T#1) and T#2) were analyzed for transcription expressions of selected genes (mRNA) or processed to single-cell suspensions without red blood cells to detect the secreted factors in the conditioned medium (cytokine/chemokine secretions). (**B**) Comparison of mRNA levels of proinflammatory chemokines (*Cxcl1* and *Ccl2*) between the LLC1 cell line and its derived tumor samples (T#1 and T#2). (**C**) Comparison of the mRNA levels of the proinflammatory chemokines (*Cxcl1* and *Ccl2*) between the TRAMP-C1 cell line and its derived tumor samples (T#1 and T#2). RT-qPCR measurements were derived from three technical replicates, and the results are presented as the mean ± SD. Student’s *t*-test. **** *p* < 0.0001. (**D**) Comparison of the cytokine/chemokine profiles of the LLC1 cell line and its derived tumors (T#1 and T#2). Differentially secreted factors are labeled with black arrows. (**E**) Comparison of the cytokine/chemokine profiles of the TRAMP-C1 cell line and its derived tumors (T#1 and T#2). Secretion of the interleukin-1 receptor antagonist (IL1RN) was increased in the tumors (white arrows).

**Figure 2:**
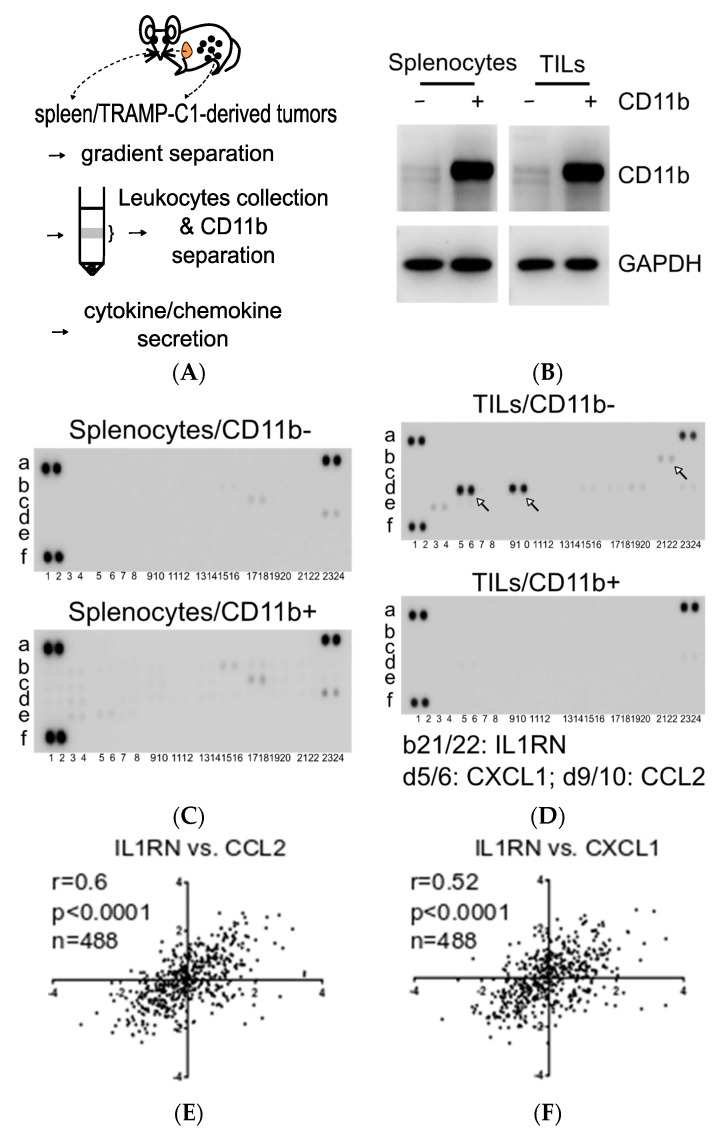
Association of interleukin-1 receptor antagonist (IL1RN) with cluster of differentiation 11b (CD11b)-deficient (CD11b^−^) tumor-infiltrating leukocytes (TILs) in the transgenic adenocarcinoma of the mouse prostate (TRAMP)-C1-derived tumor microenvironment (TME). (**A**) Outline for analyzing cytokine/chemokine profiles of leukocytes. Single-cell suspensions prepared from the spleen (orange) or tumors were separated by a Ficoll gradient to enrich leukocytes followed by CD11b-positive cell isolation using an immunomagnetic approach. Secreted factors in conditioned media from different populations were analyzed. (**B**) Characterization of CD11b-negative (−) and -positive (+) populations from splenocytes and TILs. Cell lysates were analyzed by Western blotting. (**C**) Comparison of cytokine/chemokine profiles of CD11b^−^ and CD11b^+^ populations from splenocytes. (**D**) Comparison of cytokine/chemokine profiles of CD11b^−^ and CD11b^+^ populations from TILs. Selected factors were labeled with white arrows (IL1RN, CXCL1, and CCL2). (**E**,**F**) Correlation of the human IL1RN with CCL2 and with CXCL1. mRNA expression z-scores were plotted on a log-scale. Gene expressions from a human prostate adenocarcinoma database, The Cancer Genome Atlas (TCGA) Program, were analyzed on the website cBioPortal for Cancer Genomics.

Based on the well-established syngeneic cell lines derived from the TRAMP, we identified a group of proinflammatory chemokines (e.g., CCL2) that were associated with the tumorigenic TRAMP-C1 cell line [19]. However, those chemokines were drastically reduced, both at the messenger (m)RNA and protein levels, in TRAMP-C1-derived tumors in immune-competent mice [19]. Comparison to the obviously induced expression of *Ccl2* in Lewis lung cancer cell line (LLC1)-derived tumors [20] suggested an anti-inflammatory environment in TRAMP-C1-derived tumors [19]. To address whether this inhibitory feature is specific to the TRAMP-C1-derived TME, we compared the mRNA levels and secretion profiles of the cytokines/chemokines in the TME from tumors developed in mice after subcutaneous (s.c.) injections with either the LLC1 or TRAMP-C1 cell line (Figure 1A). After comparison to the parental cell lines, the mRNA levels of the proinflammatory chemokines (*Cxcl1* and *Ccl2*) were significantly upregulated in the LLC1-derived TME (by ca. 100-fold, Figure 2B), while they were suppressed in the TRAMP-C1-derived TME (by ca. 5-fold, Figure 2C). When we monitored the cytokines and chemokines secreted into the conditioned media of the collected cells in tumors, we detected several inflammatory cytokines (i.e., interferon gamma (IFN-γ), IL-1α, and tumor-necrosis factor (TNF)-α) in the LLC1-derived TME (arrows, Figure 2D). In contrast, the natural anti-inflammatory cytokine, IL1RN, was only detected in the TRAMP-C1-derived TME, and the majority of the secreted factors were unaltered (arrows, Figure 2E). In summary, we demonstrated that the IL1RN served as a tumor-type-specific anti-inflammatory cytokine in the TRAMP-C1-derived TME.

### 3.2. Characterization of CD11b^−^ TILs Responsible for IL1RN Secretion in the TRAMP-C1-Derived TME

Our earlier studies demonstrated that TILs, not cancer cells or peripheral blood mononuclear cells in circulation, were the primary source for expressing and secreting the IL1RN in the TRAMP-C1-derived TME [19]. In light of earlier findings that in a *Pten*-null mouse prostate cancer model Gr1^+^CD11b^+^ leukocytes can secrete the IL1RN to overcome oncogene-induced senescence [13], we analyzed leukocytes prepared from either splenocytes or TRAMP-C1-derived tumors (Figure 2A,B). When we examined the cytokine/chemokine profiles in conditioned media prepared from the two populations of splenocytes, there were barely differentiated signals (Figure 2C). In contrast, the secreted patterns from the CD11b^−^ TILs (TILs/CD11b^−^) recapitulated those from the TRAMP-C1-derived TME (Figure 2D vs. Figure 1E). Unlike the condition in a *Pten*-null mouse model, our results supported that the TILs/CD11b^−^ cells, but not CD11b^+^ cells, were the main population accounting for IL1RN secretion. Since two previously defined tumor-associated proinflammatory chemokines (CXCL1 and CCL2) were also detected in addition to the IL1RN, we asked whether there was a correlation in a human prostate cancer clinical dataset. After analyzing human prostate adenocarcinomas in The Cancer Genome Atlas (TCGA) Program, the IL1RN was indeed tightly correlated with both CCL2 (Figure 2E) and CXCL1 (Figure 2F). Therefore, these results suggested that the TILs/CD11b^−^ population may play an important role in prostate cancer progression.

### 3.3. Monitoring of Proliferation Effects of Conditioned Media from TILs/CD11b^−^

**Figure 3:**
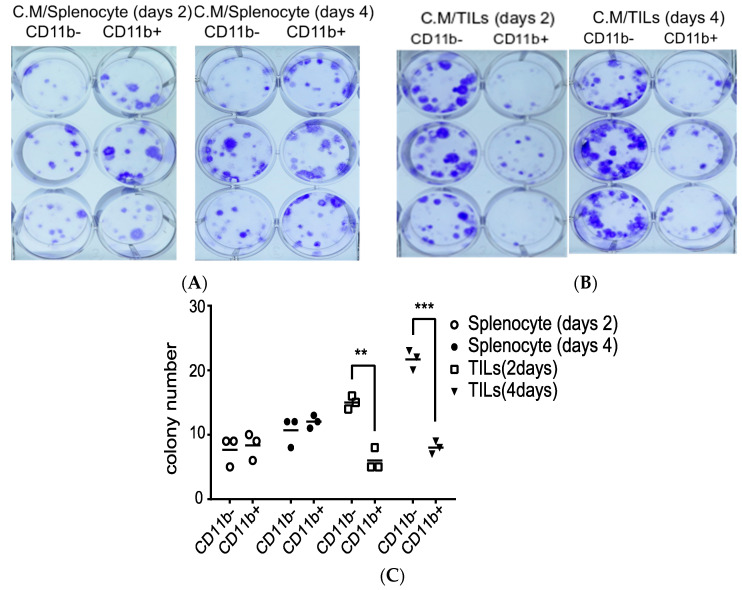
Proliferation effects of the secreted factors in conditioned media from cluster of differentiation 11b (CD11b)-deficient tumor-infiltrating leukocytes (TILs/CD11b^−^). (**A**) Colony-formation assays using conditioned media from CD11b^−^ and CD11b^+^ leukocytes enriched from splenocytes. Conditioned media were collected at different times (Days 2 and 4) and applied to transgenic adenocarcinoma of the mouse prostate (TRAMP)-C1. (**B**) Similar to panel A, conditioned media were collected from TILs enriched from TRAMP-C1-derived tumors. (**C**) Comparison of colony numbers in TRAMP-C1 cells incubated with conditioned media derived from either CD11b^−^ or CD11b^+^ leukocytes. Measurements were derived from three replicates, and results are presented as the mean ± SD. Student’s *t*-test. ** *p* < 0.01, *** *p* < 0.001.

CCL2 is involved in the malignant progression of prostate cancer [21], and the IL1RN was shown to increase the proliferation of normal prostate epithelial cells [12]. Thus, identification of a TILs/CD11b^−^ population, accounting for the cytokine/chemokine patterns observed in the TRAMP-C1-derived TME, prompted us to ask whether TILs/CD11b^−^ can facilitate tumor progression. To examine this possibility, we performed a 2D colony-formation assay to compare conditioned media from both CD11b^+^ and CD11b^−^ leukocytes. Media were collected at different times (days 2 and 4) and applied to TRAMP-C1 cells to monitor changes in colony features (e.g., size and number). As shown in Figure 3A, no apparent changes were noted when comparing the media (CD11b^−^ vs. CD11b^+^) derived from splenocytes (CM/splenocytes). However, when comparing cells cultured with the two different types of conditioned media from TILs (CM/TILs), there was clearly enhanced proliferation (in terms of increased numbers, sizes, and intensities) in cells cultured in media derived from CD11b-deficient populations (CD11b^−^ vs. CD11b^+^, Figure 3B). After calculating the colony numbers, we confirmed that the TILs/CD11b^−^ cells can increase TRAMP-C1 proliferation compared with the TILs/CD11^+^ cells, possibly via secreted factors in the conditioned media (Figure 3C).

### 3.4. Confirmation of Proliferation Effects by Tumor-Associated Proinflammatory Chemokines and the IL1RN

**Figure 4:**
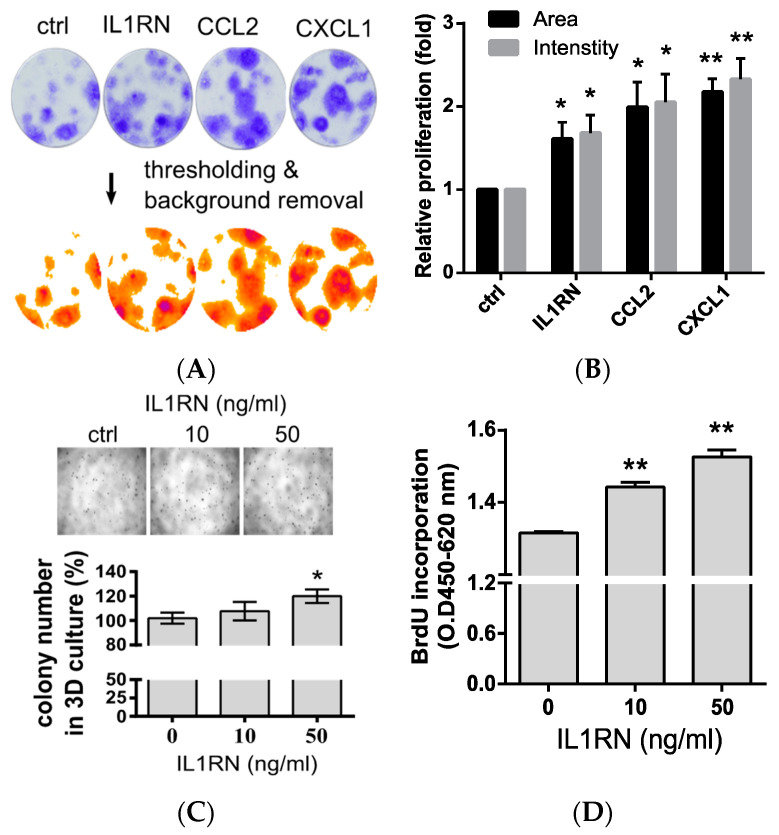
Proliferation effects of the interleukin (IL)-1 receptor antagonist (IL1RN) in transgenic adenocarcinoma of the mouse prostate (TRAMP)-C1 cells. (**A**) Colony assays using selected chemokines identified in cluster of differentiation 11b (CD11b)-negative (CD11b^−^) cells collected from TRAMP-C1-derived tumors. TRAMP-C1 cell lines were incubated with an individual chemokine (50 ng/mL). Images were processed by Image J using the *ColonyArea* plug-in [17]. (**B**) Quantification of the colony size and staining signal. Proliferation was indicated by the analyzed colony features, such as the occupied sizes (Areas) and staining signals (Intensities), from Panel (A); *n* = 3. (**C**) Monitoring of proliferation by a 3D culture approach. Proliferation effects of the IL1RN were indicated by colony numbers calculated from the images; *n* = 3. (**D**) Monitoring of DNA synthesis of TRAMP-C1 cells following IL1RN treatment. DNA synthesis was measured by BrdU incorporation. Measurements were derived from three replicates, and the results are presented as the mean ± SD. Student’s *t*-test. * *p* < 0.05, ** *p* < 0.01.

To further study the biological functions of the conditioned media from TILs/CD11b^−^ cells, we analyzed the proliferation effect using selected factors identified in the media (Figure 2D). First, we utilized a 2D colony-formation assay again to directly assess two proinflammatory chemokines (CCL2 and CXCL1) and the IL1RN. Although the proliferation effects of the exogenously added factors were not as strong as those from the conditioned media from TILs/CD11b^−^, they still showed enhanced proliferation, as evidenced by measuring the occupied areas and intensities of the colonies (Figure 4A). In general, all of the key factors identified from the TILs/CD11b^−^ media were capable of promoting proliferation (Figure 4B). Next, we focused on the effects of the IL1RN. It was shown that the IL1RN enhanced proliferation of normal prostate epithelial cells under 3D-culture conditions using physiological extracellular matrix proteins [12]. Therefore, we also examined the IL1RN effects in TRAMP-C1 cells under a 3D-culture system and found that the IL1RN increased the colony numbers (Figure 4C). Finally, we measured the DNA synthesis levels by monitoring incorporation of the deoxynucleotide analog, BrdU, in TRAMP-C1 cells. Consistent with the hypothesis that the IL1RN promotes proliferation, we observed increased BrdU incorporation after IL1RN treatment (Figure 4D). In summary, our results supported that the mouse TILs/CD11b^−^ cells and the IL1RN can promote the proliferation of TRAMP-C1 cells.

### 3.5. The IL1RN Promotes Proliferation and Suppresses the Inhibitory Effects of IL1B in Human Prostate Cell Lines

**Figure 5:**
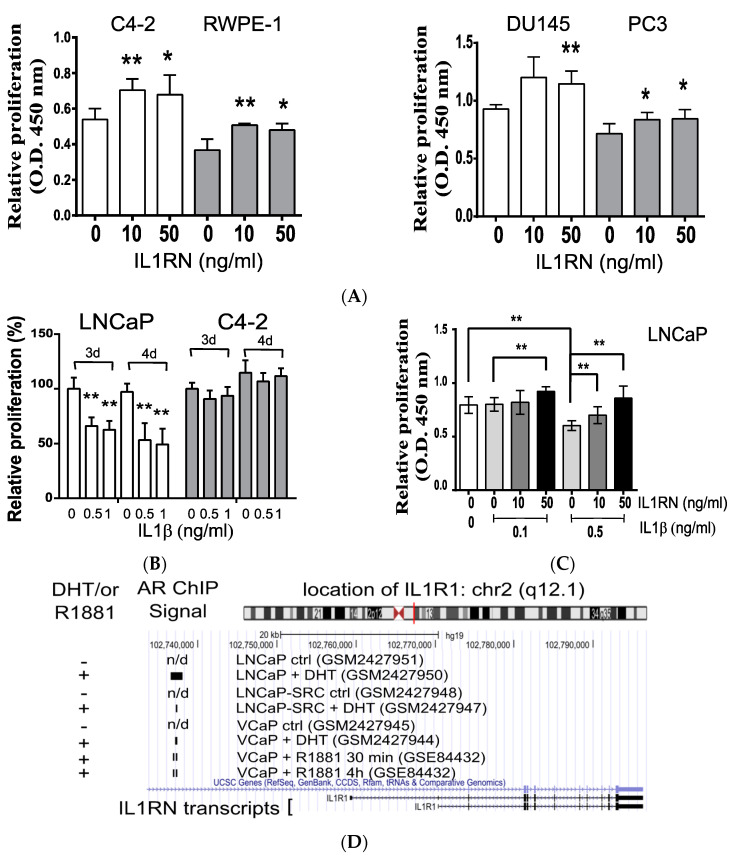

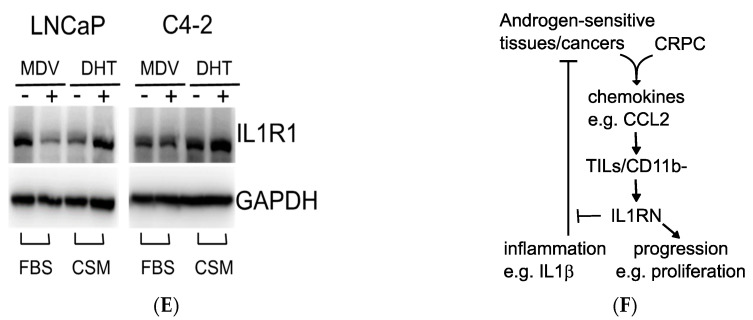
Proliferation effects of the interleukin (IL)-1 receptor antagonist (IL1RN) in human prostate cancer cell lines. (**A**) Monitoring of the proliferative effects of the IL1RN in several human prostate cell lines. Three prostate cancer cell lines (C4-2, DU145, and PC3) and one normal prostate epithelial cell line (RWPE-1) were treated with different concentration of the IL1RN for 3 days. (**B**) Monitoring of the antiproliferative effects of IL-1β (IL1β). Cells (LNCaP and C4-2) were treated with different concentrations of IL1β for different periods (3 d, 3 days; 4 d, 4 days). (**C**) Proliferation assay using the LNCaP cell line in combination of the IL1RN and IL1β. Two sets of cells were treated with a dosage range of IL1RN (0~50 ng/mL) in combination with two fixed concentrations of IL1β (0.1 and 0.5 ng/mL). (**D**) Androgen-induced recruitment of the androgen receptor (AR) to the genomic location of the IL-1 receptor, type 1 (IL1R1). Three sets of chromatin immunoprecipitation (ChIP) assays (LNCaP, LNCaP-SRC, and VCaP) in response to androgen (dihydrotestosterone (DHT) or R1881) were summarized using the UCSC Genome Browser. The GEO accession numbers are included in parentheses. The detected locations by ChIP were labeled with black bars; n/d, not detected. (**E**) IL1R1 protein levels in response to an AR inhibitor and agonist. Human prostate cancer cell lines (LNCaP and C4-2) were treated with MDV3100 (MDV) or DHT followed by a Western blot analysis. FBS, culture medium with 10% fetal bovine serum; CSM, medium with 10% charcoal-stripped serum. (**F**) A working model depicts the recruitment of cluster of differentiation 11b (CD11b)-deficient tumor-infiltrating leukocytes (TILs/CD11b^−^) by tumor-associated proinflammatory chemokines (e.g., C-C motif chemokine ligand 2 (CCL2)). TILs/CD11b^−^ cells secrete the IL1RN, which suppresses the antiproliferative effects of the inflammatory signals (e.g., IL1β) and promotes tumor progression (e.g., proliferation) of castration-resistant prostate cancer (CRPC). Student’s *t*-test. * *p* < 0.05, ** *p* < 0.01.

Since the IL1RN was involved in proliferation in the mouse TRAMP-C1 cell line, we further asked whether this effect is generally present in human prostate cancers. Although ADT is a standard procedure for recurrence after localized treatments (e.g., a prostatectomy), most patients will eventually develop CRPC [1]. Therefore, we tested the effects of the IL1RN in several CRPC cell lines. As shown in Figure 5A, we confirmed the proliferative effects of the IL1RN in three CRPC cell lines (C4-2, DU145, and PC3). In addition, it was shown that the IL1RN can promote proliferation of a normal prostate epithelial cell line (PZ-HPV-7) [12]; consistently, we demonstrated again the positive effect using another normal prostate epithelial cell line (RWPE-1) (Figure 5A). In summary, our results validated the role of the IL1RN in contributing to proliferation in both normal and CRPC human prostate cell lines.

Deprivation of mitogenic stimuli during ADT evokes an inflammatory environment [3,4,5]. It is possible that cellular responses to inflammatory signaling differ between androgen-sensitive prostate tissues and CRPC. Indeed, we showed that the androgen-dependent LNCaP cells were more sensitive to the cytotoxic effects of IL1β than was the C4-2 CRPC cell line (Figure 5B). Therefore, the results suggested that the IL1RN secreted by TILs/CD11b^−^ may be involved in protecting both host tissues and tumor cells from inflammation-induced cytotoxic effects. Indeed, the IL1RN could rescue LNCaP cells from the inhibitory effects of IL1β (0.5 ng/mL) in a concentration-dependent manner (Figure 5C). Since only LNCaP, but not C4-2 cells, were sensitive to IL1β, and both IL1β and the IL1RN compete with the same receptor, IL1R1, we investigated the levels of IL1R1 in response to different AR activities. Based on several published genome-wide analyses of chromatin immunoprecipitation, physical associations of the AR near transcription locations of the IL1RN were dependent on androgens (Figure 5D), supporting transcriptional regulation by the AR. Furthermore, the protein levels of IL1R1 were tightly correlated with AR activities in the androgen-sensitive LNCaP, but not the C4-2 cell line (Figure 5E). Treatment with an AR activator (DHT) increased the IL1R1 levels while an inhibitor (MDV) reduced those levels in LNCaP cells (Figure 5E). Therefore, the homeostasis of normal prostate tissues and androgen-sensitive prostate cancers might be established by the driving force of androgen signaling and the inhibitory functions of inflammatory signaling, such as IL1β/IL1R1. In summary, we demonstrated that the IL1RN promotes proliferation of CRPC and might protect androgen-sensitive cells from the cytotoxic effects activated by inflammatory signals.

## 4. Discussion

In this study, we found that the IL1RN was associated with the TRAMP-C1-derived TME, which exhibited an anti-inflammatory environment. We demonstrated that TILs with a cluster of differentiation 11b (CD11b)-deficient population (TILs/CD11b^−^) were responsible for IL1RN secretion in the TME; in addition, conditioned media from the TILs/CD11b^−^ cells promoted proliferation. The proliferative functions of the recombinant IL1RN protein were confirmed in mouse TRAMP-C1, human CRPC (C4-2, PC3, and DU145), and normal epithelial (RWPE-1) cell lines. Unlike resistance shown in the C4-2 CRPC cell line, androgen-sensitive LNCaP cells were suppressed by IL1β/IL1R1 signaling, which could be rescued by the IL1RN. The inhibitory effects of IL1β and the positive correlation of IL1R1 levels with AR activities in LNCaP, but not C4-2 cells, suggested an interplay between the inflammation and androgen signals in maintaining the homeostasis of androgen-sensitive prostate tissues and cancers. Thus, a simplified model for determining the life-or-death outcomes of these types of cells is based on a summation of the two opposite signals: androgen/AR/IL1R1 (life) and IL1β/IL1R1 (death). Regular levels of the IL1RN may be sufficient to counteract the inhibitory signaling of the IL1β/IL1R1 pathway in normal prostate tissues; however, in the early stages of androgen-sensitive prostate cancers, cells with mutations that lead to increasing AR activities (e.g., amplification) consequently elevate IL1β/IL1R1 signaling, which might outweigh the IL1RN’s functions, and thus they become sensitive to inflammation-induced cytotoxicity. Our results suggested that CRPC is resistant to the inhibitory effects of inflammation (Figure 5B); therefore, therapeutic strategies targeting AR functions (e.g., ADT) selectively remove the androgen-sensitive prostate cells and might enrich inflammation-resistant CRPC. Based on our results, we propose a working hypothesis that prostate cancers recruit the TILs/CD11b^−^ populations that secrete the IL1RN, which ameliorates the inflammatory stresses to androgen-sensitive cancers. The IL1RN in the TME might further support the clonal expansion of CRPC (Figure 5F).

The transition of androgen- and inflammation-sensitive cancers to CRPC is consistent with the immunoediting theory, which consists of three major phases of elimination, equilibrium, and escape [22]. ADT is initially efficient in treating prostate cancers; however, complex inflammatory signals established in the TME, as evidenced by many kinds of leukocytes [3,4,5], eventually select for inflammation-insensitive CRPC. It is possible that the IL1RN is induced by TILs under stress responses to ADT. Supporting this hypothesis, in an earlier study we once compared the mRNA levels of the IL1RN before ADT (pre-ADT) and after ADT (post-ADT, 22 weeks after ADT) in seven prostate cancer patients [23]. After analysis, the average levels of the IL1RN were found to be higher in patients after ADT than those before ADT, consistent with the ADT-induced expression of the IL1RN [19]. Furthermore, the roles of IL1β seem to be more direct in facilitating the transition from androgen-sensitive cancers to CRPC than the elimination processes of the immunoediting theory. It was shown that IL1β can convert several types of AR antagonists (e.g., bicalutamide) to agonists through modifying the inflammatory sensor, TAB2, in the nuclear receptor corepressor 1 (N-CoR) complex [5]. Thus, inflammatory signals not only select for resistant cells, but also contribute to cellular mechanisms leading to the development of CRPC. It is not clear how different the dosage responses are to IL1β between its induced cytotoxicity and CRPC transition in androgen-dependent prostate cancers in vivo. However, IL1RN secretion by TILs during ADT might provide a TME favoring CRPC development at certain tumor stages.

Although IL1β and the IL1RN are known to compete with the same receptor, IL1R1, functions of the IL1RN might not be restricted to counteracting IL1β-related functions. Our earlier studies showed that the IL1RN was capable of inhibiting the induction of proinflammatory chemokines in response not only to IL1β but also to TNF-α [19], suggesting that the IL1RN is involved in signal pathways other than IL1R1-mediated function. Earlier studies also showed that IL1RN treatment activates mitogenic signaling [12]; however, it is not yet clear whether these results are due to crosstalk between IL1R1 signaling and other pathways. Another key issue is the identity of the IL1RN-secreting cells. Earlier studies showed that the Gr1^+^/CD11b^+^ cells derived from the TME in a *Pten* null mouse prostate model secrete the IL1RN and antagonize oncogene-induced senescence [13]. However, our results showed that the IL1RN-secreting cells in the TRAMP-C1-derived TME were CD11b-deficient TILs (Figure 2D). Interestingly, another study working on inflammatory stimuli in a pancreatic intraepithelial neoplasia model found that Ym1^+^ macrophages released both the IL1RN and CCL2, which contributed to fibrogenesis and tumorigenesis [14]. Since the likely human homolog of Ym1 is acidic mammalian chitinase (gene name: *CHIA*) [24], it would be interesting to determine the function of CHIA-positive TILs in tumor biology. However, when we analyzed gene expressions in several human prostate cancer datasets, the identified groups with higher mRNA levels of the three genes (IL1RN, CD11B, and CHIA) did not overlap (mRNA High, Figure S2). Since Ym1^+^ macrophages were identified in a pancreatic mouse model, it is not clear whether the same group of cells is important in prostate cancer models. Further investigations are warranted to understand the role of IL1RN-secreting TILs in tumor biology.

## 5. Conclusions

We found that CD11b-deficient, tumor-infiltrating leukocytes secrete IL1RN, which is crucial for tumor progression of prostate cancers. 

## Supplementary Materials

The following are available online at https://www.mdpi.com/2227-9059/8/12/602/s1, Figure S1: Quantification of colonies in 3D culture approach. Figure S2: Gene expression patterns in three prostate cancer databases, Table S1: Primer sequences for the RT-qPCR. Table S2: Abbreviations used in the article.
