# Supplementary material for: Roles of Interleukin-1 Receptor Antagonist in Prostate Cancer Progression
Source: Biomedicines. 2020 Dec 13;8(12):602. doi: 10.3390/biomedicines8120602 (PMC7763916; doi:10.3390/biomedicines8120602)
Supplement: Supplementary file 1 [file biomedicines-08-00602-s001.pdf]

Roles of interleukin-1 receptor antagonist in prostate cancer progression

Yu-Ching Fan, Kuan-Der Lee, Yuan-Chin Tsai

Supplementary Figure S1

Quantification of colonies in 3D culture approach.

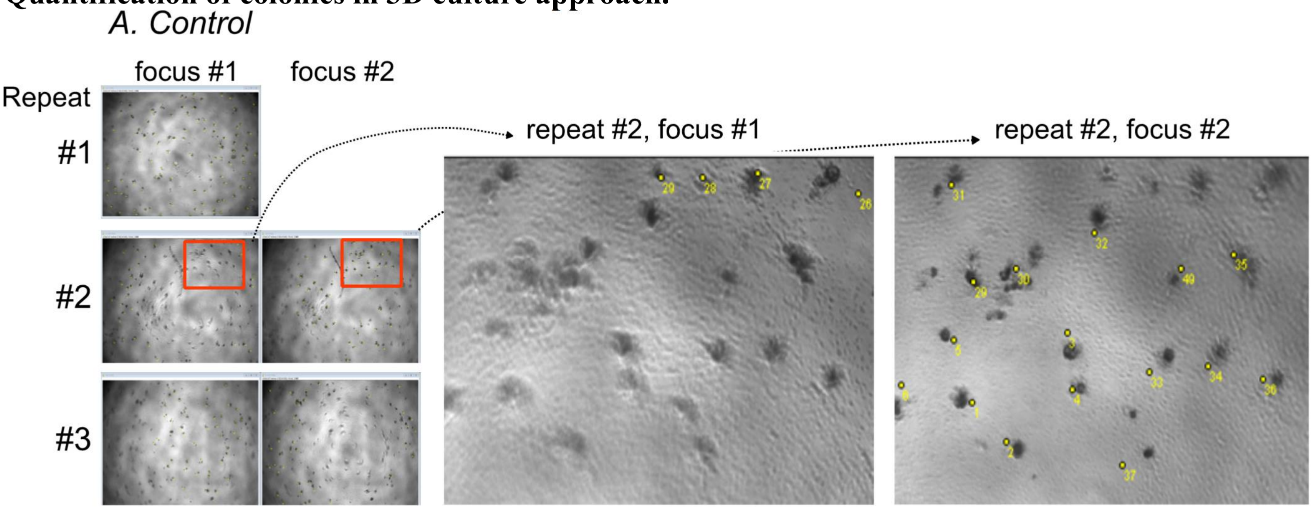

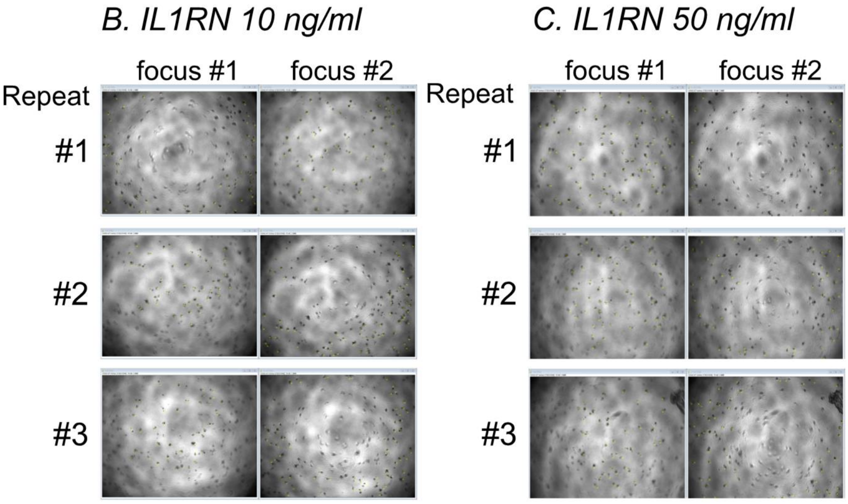

D. Summary of colonies

Treatment	Repeat	Cell counting by ImageJ	Total counts
Ctrl	1	104	104
	2	51+42	93
	3	47+50	97
IL1ra (10 ng/ml)	1	51+52	103
	2	47+72	119
	3	44+67	111
IL1ra (50 ng/ml)	1	87+39	126
	2	59+63	122
	3	47+78	125

Figure legend

(A-C) TRAMP-C1 cells were seeded on Matrigel-coated wells and treated with different concentration of IL1RN (control, panel A; 10 ng/ml, panel B; 50 ng/ml, panel C). Each condition was performed in triplicate (Repeat #1-#3). The images of technical repeats were taken with different focal planes and two selected images (focus #1, focus #2) were used for quantification. Colonies identified in the two focal planes were not overlapped (as demonstrated with the two red boxes) and then counted using Image J software. (D) The identified colonies in two focal planes were combined to derive the total counts in each repeat.

Supplementary Figure S2

Gene expression patterns in three prostate cancer databases.

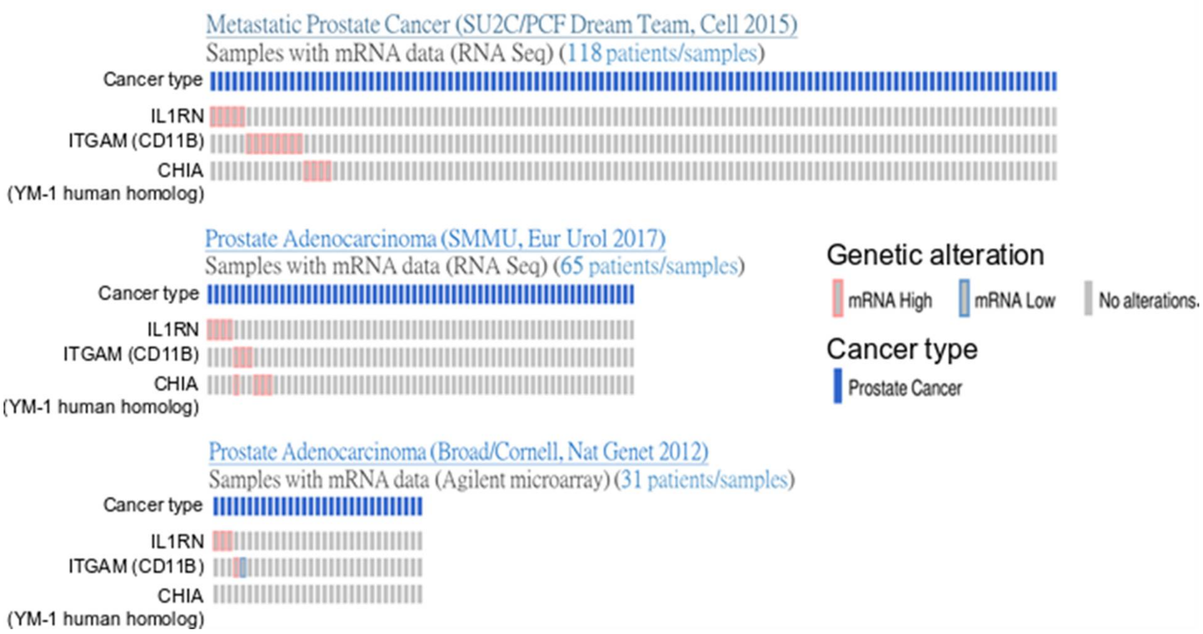

Figure legend

A bar code plot for mRNA expression of IL1RN, ITGAM (CD11b), and CHIA (human homolog gene of mouse YM-1) in three different prostate cancer clinical databases were analyzed using the cBioPortal website.

Supplementary Table S1. Primer sequences for the qRT-PCR.

Gene	5'-3'
IL1RN F	CAG CTC ATT GCT GGG TAC TT
IL1RN R	CTC AGA GCG GAT GAA GGT AAA G
CXCL1 F	GTG TCT AGT TGG TAG GGC ATA AT
CXCL1 R	CAG TCC TTT GAA CGT CTC TGT
CCL2 F	AGT AGG CTG GAG AGC TAC AA
CCL2 R	GTA TGT CTG GAC CCA TTC CTT C
GAPDH F	TCT CCC TCA CAA TTT CCA TCC
GAPDH R	GGG TGC AGC GAA CTT TAT TG

Supplementary Table S2. Abbreviations used in the article

Abbreviation	Full name
ADT	Androgen-deprivation therapy
AR	Androgen receptor
BrdU	Bromodeoxyurea
CCL2	C-C motif chemokine ligand 2
CRPC	Castration-resistant prostate cancer
CD11b	Cluster of differentiation 11b
CHIA	Acidic mammalian chitinase
DHT	Dihydrotestosterone
ELISA	Enzyme-linked immunosorbent assay
IFNγ	Interferon gamma
IL1α	Interleukin-1 alpha
IL1β	Interleukin-1 beta
IL1RAP	Interleukin-1 receptor accessory protein
IL1RN	Interleukin-1 receptor antagonist
IL1R1	Interleukin-1 receptor type 1
LLC1	Lewis lung carcinoma
LNCaP	Lymph node carcinoma of the prostate
MDV	AR inhibitor MDV3100
N-CoR	Nuclear receptor corepressor 1
RT-qPCR	Reverse-transcription quantitative polymerase chain reaction
TCGA	The cancer genome atlas
TILs	Tumor-infiltrating leukocytes
TME	Tumor microenvironment
TNFα	Tumor necrosis factor alpha
TRAMP-C1	transgenic adenocarcinoma of the mouse prostate cell line
